# MicroRNA‐155 (miR-155) as an accurate biomarker of periodontal status and coronary heart disease severity: a case–control study

**DOI:** 10.1186/s12903-023-03584-w

**Published:** 2023-11-16

**Authors:** Zina A. Daily, Batool Hassan Al-Ghurabi, Ahmed Makki A. Al-Qarakhli, Ryan Moseley

**Affiliations:** 1 Department of Periodontics, College of Dentistry, University of Baghdad, Baghdad, Iraq; 2 Department of Periodontics, College of Dentistry, University of Al-Ameed, Karbala, Iraq; 3 Department of Basic Science, College of Dentistry, University of Baghdad, Baghdad, Iraq; 4 Department of Oral Diagnosis, College of Dentistry, University of Anbar, Ramadi, Iraq; 5 Disease Mechanisms Group, School of Dentistry, College of Biomedical and Life Sciences, Cardiff University, Cardiff, UK

**Keywords:** Periodontitis, Coronary heart disease, MicroRNA‐155, Inflammation, Interleukin-1β, Biomarker

## Abstract

**Background:**

Increasing evidence supports associations between periodontal disease and coronary heart disease (CHD). This case–control study evaluated whether inflammatory regulator, microRNA-155 (miR-155), could be utilised as a biomarker of periodontitis and/or CHD.

**Methods:**

Of 120 participants, 30 patients had clinically healthy periodontium (controls, C), 30 patients had generalized periodontitis (P), 30 patients had CHD and clinically healthy periodontium (AS-C); and 30 patients had CHD with generalized periodontitis (AS-P). Patient demographic and periodontal characteristics (plaque index, bleeding on probing, probing pocket depth and clinical attachment loss), were collected. Patient whole blood and saliva levels of miR-155 and pro-inflammatory cytokine (interleukin-1β), were quantified by quantitative real time polymerase chain reaction (qRT-PCR) and enzyme-linked immunosorbent assay (ELISA). One-way ANOVA with post-hoc Tukey test was used to determine differences among the four groups. Chi Square test was used for participant gender comparisons. Pearson correlation tests and multiple linear regression analyses were used to assess associations between the demographic and clinical variables analysed, versus IL-1β and miR-155 levels. miR-155 and IL-1β accuracy in differentiating healthy versus other patient groups were analysed using receiver operating characteristic (ROC) curves, by calculating area under the curve (AUC) values and sensitivity and specificity cut-off points using Youden’s index. Statistical tests of sensitivity and specificity were conducted using the McNemar test.

**Results:**

Whole blood miR-155 levels were elevated in periodontitis/non-periodontitis patients with CHD (AS-P, AS-C), and periodontitis patients alone (P) (*p* < 0.001). Receiver operating characteristic (ROC) and area under the curve (AUC) analyses confirmed miR-155 accuracy in discriminating P, AS-C and AS-P groups (AUC 0.6861–0.9944, *p* < 0.0001–0.05), coupled with high sensitivity (76.7–100.0%), specificity (53.3–96.7%) and cut-off points (> 0.955- > 2.915 a.u.; *p* < 0.0001). miR-155 levels further distinguished between CHD (AS-C, AS-P) and periodontitis (P) patients (AUC ≥ 0.8378, sensitivity ≥ 88.7%, specificity ≥ 73.3%, cut-off > 2.82 a.u; *p* < 0.0001), and between AS-C and AS-P patients (AUC 0.7578, sensitivity 80.0%, specificity 50.0%, cut-off > 7.065 a.u; *p* < 0.001). Subsequent analyses identified positive correlations between miR-155 and the various patient demographics, salivary interleukin-1β and periodontal parameters assessed.

**Conclusions:**

This study advocates miR-155 as an accurate diagnostic/prognostic biomarker of periodontitis and/or CHD severity, thereby improving detection and treatment for both conditions.

**Supplementary Information:**

The online version contains supplementary material available at 10.1186/s12903-023-03584-w.

## Background

Periodontal diseases, comprising gingivitis, periodontitis, mucositis, gingival enlargements, and peri-implantitis, typically describe a range of chronic inflammatory conditions affecting tissues of the periodontium. These diseases are regarded as the most common disease in Man, leading to huge economic burdens for healthcare systems worldwide [1]. Dental plaque accumulation and uncontrolled bacterial biofilm formation, predominantly composed of pathogenic Gram-negative bacterial species, such as *Porphyromonas gingivalis*, are commonly associated with the initiation of periodontitis [2, 3]. However, periodontitis is now regarded as a chronic inflammatory condition borne out of dysbiosis within the periodontal microbiome, leading to the loss of supporting connective tissues via dysregulated host-derived mechanisms, with immuno-inflammatory responses to sub-gingival biofilms being key determinants of disease severity and progression [4–6].

In addition to the direct impact on oral tissues, there is growing evidence to support significant associations between periodontal disease and other chronic inflammatory conditions, including atherosclerosis and coronary heart disease (CHD) [7]. Coronary artery stenosis or occlusion results in CHD, which has a multitude of life-threatening symptoms linked to hyper-stimulated vascular immuno-inflammatory responses that contributes to the onset and progression of CHD and associated pathologies. Epidemiological evidence strongly supports an association between periodontitis and atherosclerosis/CHD [8–11], with periodontitis proposed to exacerbate these conditions via enhanced periodontal pathogen circulation and systemic inflammation [10, 12]. The impact of periodontal therapy interventions on cardiovascular outcomes have further been explored, with treatments shown capable of significantly reducing pro-inflammatory biomarker levels, such as C-reactive protein (CRP), interleukin-1β (IL-1β) and IL-6, and systemic inflammation overall [13, 14]. However, although hereditary factors play roles in the development of periodontitis and CHD, it remains unclear how genetic predispositions influence the excessive inflammation and elevated pro-inflammatory mediators associated with these diseases [15–17]. Thus, despite numerous studies attempting to identify specific loci or genes associated with the underlying immuno-inflammatory mechanisms and correlations between these diseases, these have remained elusive.

In recent years, microRNA (miR) research has increasingly emerged, with investigations into the development of novel miR-based, diagnostics and therapeutics for disease situations, including periodontitis and CHD [18–21]. miRs are small, highly conserved non-coding RNA molecules involved in the epigenetic regulation of gene expression via the promotion of mRNA degradation or repression of mRNA translation, which regulate numerous cellular processes. miRs are transcribed by RNA polymerases II and III, generating precursors that undergo cleavage to form mature miRs [22]. In light of their stability in human biofluids, circulating miRs are considered promising diagnostic and prognostic biomarkers of disease, whilst the translation development of miR-based therapies is also acknowledged to possess huge potential in the treatment of various diseases. miRs serve several functions within the oral cavity, including the regulation of the immuno-inflammatory responses during periodontal disease [23–25]. Similarly, miRs are further established to regulate plaque formation that leads to CHD, correlating with significant changes in inflammation, angiogenesis and apoptosis [26, 27].

miR-155 is a microRNA encoded on chromosome 21, located in the 3' untranslated region (3UTR) in humans. It plays a critical role in regulating multiple biological processes, such as cell proliferation, differentiation and apoptosis [22]. As miR-155 is also established to exhibit both pro- and anti-inflammatory properties, it is regarded as a master regulator of inflammatory responses, with chronic inflammatory diseases associated with aberrant miR expression [28]. Thus, miR-155 has received attention as a potential salivary or crevicular fluid biomarker of active inflammatory periodontitis alone or concomitant with additional underlying co-morbidities, such as diabetes mellitus [29–31]. Similarly, numerous studies have demonstrated that miR-155 levels correlate with CHD severity [32–34]. Therefore, as miR-155 has been proposed as a potentially viable biomarker of inflammation and disease activity, the primary objective of this study was to accurately quantify whole blood miR-155 expression and salivary pro-inflammatory cytokine, interleukin-1β (IL-1β), protein levels in generalized periodontitis patients, with or without additionally diagnosed CHD, versus periodontal healthy patient control groups, with or without diagnosed CHD. The secondary objective was to determine whether correlations existed between miR-155 and IL-1β levels and the extent of patient periodontitis, either alone or in association with CHD.

## Materials and methods

### Study design

This study was designed as a case–control study. The protocol for the study was approved by the Ethics Committee of the University of Baghdad, College of Dentistry (number 652622). The study was conducted at the Department of Periodontics, Al-Ameed University Dentistry College and Hospital, the Karbala Centre of Specialised Dentistry, the Imam Al-Hussain Medical City; and the Karbala Centre of Cardiovascular Diseases and Surgery, between April–October 2022.

### Sample size calculation

Based on the prevalence of periodontitis and CHD based on existing evidence [7, 35], a pilot study was originally conducted, where the initial six whole blood samples collected for each Group were quantified for miR-155 levels, as described below. The miR-155 levels obtained were subsequently used to calculate the sample size required, according to the following formula [36]:-

$$\mathrm{Sample\hspace{0.17em}size}\hspace{0.17em}=\hspace{0.17em}\mathrm{r}\hspace{0.17em}+\hspace{0.17em}1/\mathrm{r}\hspace{0.17em}\times \hspace{0.17em}(\mathrm{SD})2\hspace{0.17em}\times \hspace{0.17em}(\mathrm{Z\beta }\hspace{0.17em}+\hspace{0.17em}\mathrm{Z\alpha }/2)2/\mathrm{d}2$$

where r (ratio of cases to controls) = 2; SD is the standard deviation (calculated as 58.613); Zβ is the standard normal variate for power of 80% (calculated as 0.84); Zα/2 is a 5% type 1 error (calculated as 1.96); and d is the expected mean difference between cases and controls (calculated as 18.462). Therefore, the required sample size for each Group was calculated as 79, which was rounded up to 90 to address any potential patient withdrawal from the study. Accordingly, each study and control Group received 30 subjects, resulting in a total number of 120 participants enrolled in the study, at a test power of 80% and α probability of 0.05.

### Patient inclusion and exclusion criteria

General inclusion criteria for recruited patients included (a) adults, aged 35–75 years; (b) body mass index (BMI) < 25kg/m^2^; (c) possession of ≥ 20 teeth present without caries or crowns; and (c) in good overall health, with no other systemic comorbidities other than CHD in the cardiovascular disease-related groups. Regarding periodontal diagnoses, exclusion criteria included patients (a) with systemic diseases that may affect the development or progression of periodontal disease, (b) receiving periodontal treatment in the last 6 months, (c) who were smokers or had other behavioural variables, (d) who had taken corticosteroids or antibiotics in the last 3 months; and (e) who were pregnant or nursing at the time of the study, (f) who had dentures, dental implants or previous periodontitis with the molar-incisor pattern. Relating to CHD diagnoses, exclusion criteria included patients (g) who had heart failure, (h) thrombocytopenia (< 100 × 10^9^ platelets/L) or anaemia (hemoglobin < 10 g/dL), (i) who had any chronic illnesses, such as cancer, liver cirrhosis or end-stage renal failure; and (j) who had a history of hemorrhagic disorder, stroke or gastrointestinal ulcer.

### Study participants

Prospective patients (*n* = *1,370*) were initially screened to assess their eligibility for recruitment onto the study overall. However, of these, certain patients did not consent to study participation (*n* = *387*), whilst others were excluded from participation (*n* = *863*), as they were smokers or blood tests revealed systemic diseases, such as diabetes mellitus, hypertension or anaemia. Participants meeting the inclusion criteria described above were either periodontitis patients requiring treatment and/or patients requiring cardiac therapy for CHD. The demographic information of the participants, such as age, gender and BMI, were collected. All participant signed an informed consent form following provision of detailed information about the study and its purpose in a consecutive series manner. All research protocols and data collection performed followed the principles outlined in the Helsinki Declaration, as revised in 2013.

Based on the sample size calculations above, the selected participants were classified into four groups based on their periodontal and CHD clinical diagnoses: (1) 30 patients with clinically health periodontium served as the control group (C), (2) 30 patients with generalized periodontitis (P), (3) 30 patients with CHD and clinically healthy periodontium (AS-C); and (4) 30 patients with CHD with generalized periodontitis (AS-P). Participants in the systemically healthy Control group (C), possessed a clinically intact periodontium with no signs of gingival inflammation, as bleeding on probing (BOP) < 10%, probing pocket depth (PPD) ≤ 3 mm, and no clinical attachment loss (CAL) [37, 38]. In addition, the periodontitis groups (P and AS-P), were defined in line with the 2017 classification of periodontal diseases and conditions, with participants having generalized periodontitis in ≥ 30% of teeth, detectable interdental CAL in ≥ 2 non-adjacent teeth, or detectable buccal or oral CAL of ≥ 3 mm, and PPD > 3mm in ≥ 2 teeth [37, 38]. Periodontitis cases also exhibited stages III or IV and grade B or C, unstable status (PPD ≥ 5 mm or PPD ≥ 4 mm with BOP), with no additional risk factors (such as diabetes mellitus and/or smoking).

**Fig. 1:**
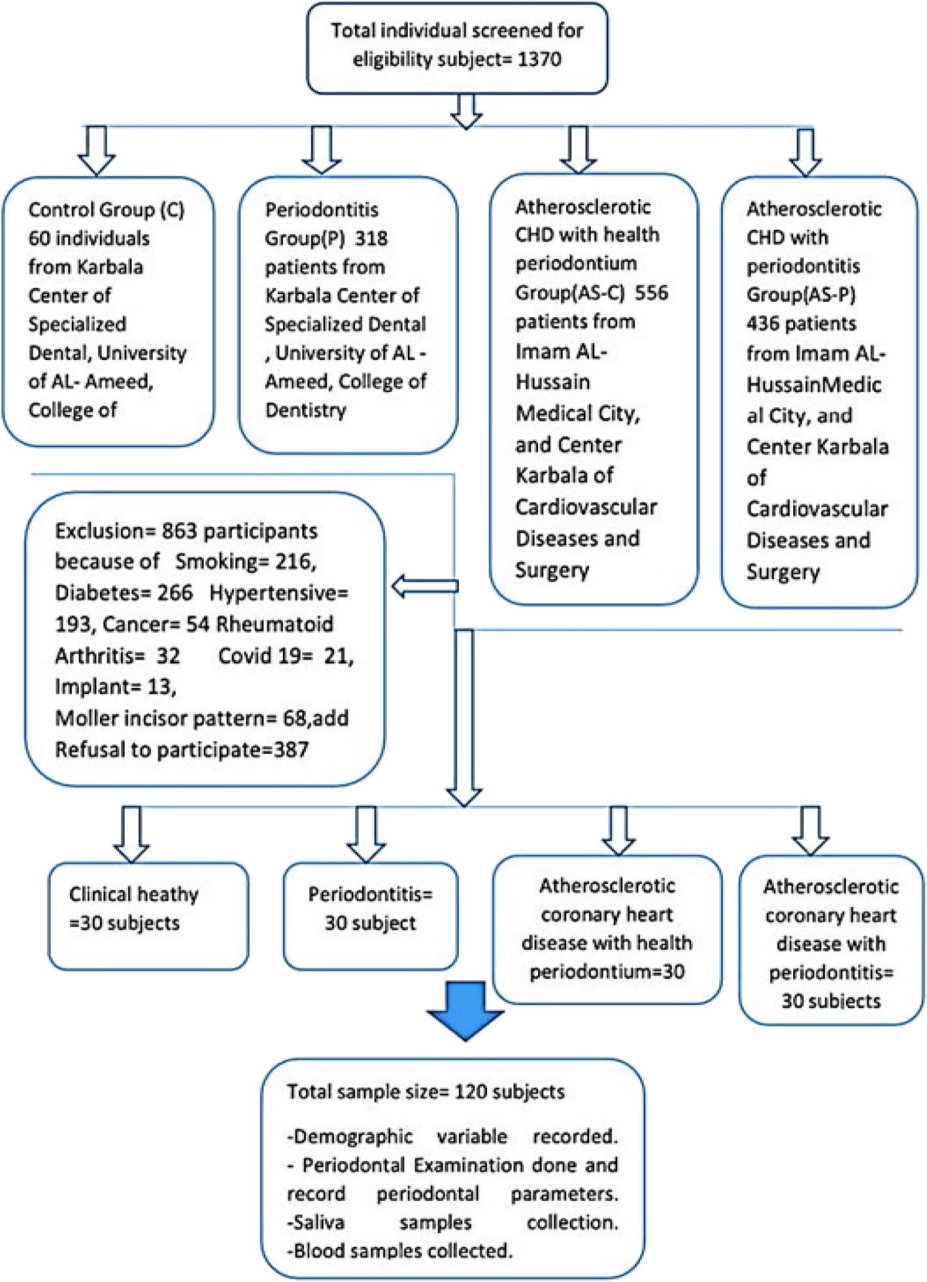
Flowchart detailing the complete study design and selection criteria

All CHD-related diagnoses were performed by a Specialist Cardiologist. Participants in the CHD groups (AS-C and AS-P) were diagnosed based on the classical clinical findings, such as chest discomfort/pain, dyspnea, tachycardia, electrocardiogram (ECG) changes [non-ST-elevation myocardial infraction-heart attack (NSTEMI) or ST-elevation myocardial infraction-heart attack (STEMI)]. Further noninvasive coronary screening using Computerized Tomography Angiography confirmed CHD diagnoses, with increased atherosclerotic plaque lesion size (stenosis) by > 50% in one or more coronary arteries, as diagnosed by cardiac catheterisation and in accordance with previously reported studies criteria [32, 39–42]. In addition, these patients also had elevated blood cardiac marker levels, such as troponin, total cholesterol, low-density lipoprotein (LDL), triglycerides, with decreased high-density lipoprotein (HDL) levels, following laboratory analyses. Further study design details and patient selection criteria are shown in Fig. 1.

### Periodontal screening, calibration and examination

Sample site evaluations were used to clinically assess periodontal parameters for all teeth presented, except wisdom teeth, involving full mouth plaque index (PLI). The percentage PLI scores at four surfaces were also recorded, as surface plaque present per total number of the examined surfaces, multiplied by 100 [43]. Full mouth BOP, the percentage of bleeding occurring at 6 sites for each tooth (mesiofacial, facial, distofacial, mesiolingual, lingual, and distolingual regions) were recorded, as the number of surface with bleeding on probing per total number of the examined surfaces, multiplied by 100 [44]. PPD were recorded as the number of graduation of probe from the gingival margin to the base of pocket, whilst CAL were recorded as the number of graduation of probe from the cementoenamel junction to the base of pocket, using a periodontal probe (PCPUNC 15; Hu-Friedy, Chicago, USA). PPD and CAL levels were determined at 6 sites for each tooth.

Prior to study commencement, a trained investigator examined the periodontal regions of all participants at the Department of Periodontology, College of Dentistry and Hospital, University of Al-Ameed. To limit intra‐examiner variability, clinical periodontal parameter data were recorded twice within an hour of examining non‐study sample patients with generalized periodontitis (*n* = *5*). The average k‐values were calculated to be 0.89 for BOP and 0.91 for PLI. The intra-class correlation coefficients (ICC) for PPD and CAL were 0.94 and 0.88 respectively, confirming measurement reliability.

### Saliva sampling and IL-1β quantification

All participants were encouraged to practice good oral hygiene regimen throughout the study duration. Prior to oral examination, unstimulated whole saliva samples were collected from study subjects using the passive drool saliva collection method, between 0900–1200. Each subject was provided with a plastic cup and asked to let the saliva flow into the cup, without any external stimulation or spitting for 5 min. A micropipette was used to transfer aliquots (300 μL) of each collected saliva sample into Eppendorf tubes for centrifugation (3,000 rpm, 20 min; ThermoFisher Scientific, Waltham, USA), prior to storage at -20 °C. Salivary IL-1β levels were subsequently quantified by ELISA (MyBioSource, San Diego, USA), according to manufacturer’s instructions.

### Whole blood collection and miR-155 quantification

Venous blood (approximately 2 mL) was collected from each study participant under aseptic conditions and transferred into tubes containing TRIzol™ Reagent (ThermoFisher Scientific). Tubes were shaken for 1 min and stored at -40 °C, until required. Total RNA extraction was performed for each blood sample using an RNA Isolation Kit-Blood (Promega, Madison, USA), according to the manufacturer's instructions. RNA was quantified by the spectrophotometric measurement of 260:280 nm ratio, with accepted values > 1.8. Reverse transcription polymerase chain reactions (RT-PCR) were performed using total RNA (5 μL) and a GoScript™ Reverse Transcription Kit (Promega), per manufacturer’s protocols. Complementary DNA (cDNA) was subsequently produced and quantified (QuantiFluor^®^ dsDNA System, Promega), according to manufacturer’s instructions. Each reaction was prepared in a total volume of 10 μL, consisting of DNA (1 μL, 1 ng/μL), Power SYBR Green Master Mix (5 μL, ThermoFisher Scientific), nuclease-free water (3 μL); and the following forward and reverse primers (0.5 μL each): GTTGGCTCTGGTGCAGGGTCCGAGGTATTCGCACCAGAGCCAACTGTTAA (miR-155-3p-RT) and GTTTGGCTCCTACATATTAGCA (miR-155-3p-F). The small nucleolar RNA, RNU43, was employed as the housekeeping gene: GTTGGCTCTGGTGCAGGGTCCGAGGTATTCGCACCAGAGCCAACAATCAG (RNU43-RT) and GTGAACTTATTGACGGGCG (RNU43-F). Quantitative RT-PCR (qRT-PCR) was performed using a Mic qPCR Cycler (Bio Molecular System, Dural, Australia). Reaction conditions had a denaturing step of 70 °C (5 min), annealing at 25 °C (5 min), extension at 42 °C (1 h), and denaturation at 70 °C (15 min). Relative fold changes in miR-155 expression (RQ) were calculated for all groups using the 2^–ΔΔCt^ method [45], normalised to the RNU43 housekeeping gene.

### Statistical analysis

Statistical analyses were performed using SPSS^®^ Statistics, Version 22 (IBM, Chicago, USA). Prior to analysis, the Gaussian distribution of the data was determined using the Shapiro–Wilk test of normality, which indicated that data obtained for the miR-155 expression levels and IL-1β protein levels were normally distributed. All data parameters were expressed as mean ± standard deviation (SD) and statistically compared using one-way Analysis of Variance (ANOVA), with post-hoc Tukey test. Statistical comparisons relating to participant gender were evaluated using a Chi Square test. Pearson correlation tests were used to assess associations between the demographic and clinical variables analysed, versus salivary IL-1β protein levels and whole blood miR-155 expression levels to analyse the influence of interaction between these variables on the levels of the markers studied, with additional linear regression analyse to confirm the association between whole blood miR-155 expression levels and salivary IL-1β protein levels. To evaluate the accuracy of miR-155 expression and IL-1β protein levels in differentiating healthy from generalized periodontitis patients with and without CHD, receiver operating characteristic (ROC) curves were constructed by calculating the area under the curve (AUC) values and identifying cut-off points to estimate sensitivity and specificity, as assessed by the Youden’s index. Statistical tests of sensitivity and specificity were conducted using the McNemar test for the correlated proportion of IL-1β and miR-155 levels in patients with elevated risk for periodontitis and CHD, including a 95% CI. This study did not contain any indeterminate or missing data. Significance was considered at p < 0.05.

## Results

### Patient demographics and periodontal parameters

**Fig. 2:**
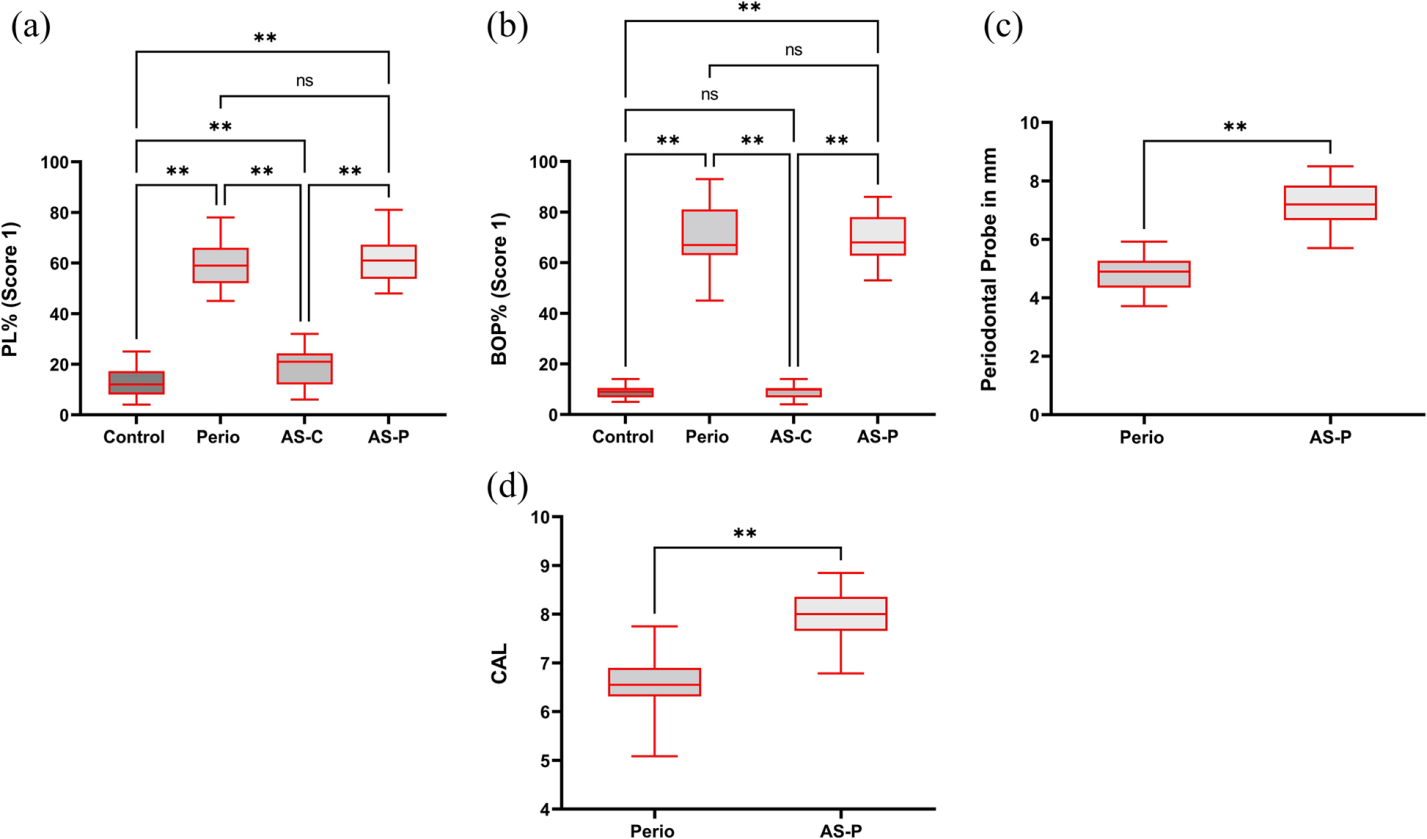
Comparison of clinical periodontal parameter data between the control (C), generalized periodontitis (P), CHD and clinically healthy periodontium (AS-C); and CHD with generalized periodontitis (AS-P) groups, for mean patient (**a**) PLI scores, (**b**) BOP scores, (**c**) PPD values, and (**d**) CAL values. Data are expressed as the mean ± SD. Comparisons made using one-way Analysis of Variance (ANOVA), with post-hoc Tukey test. **p < 0.001, ns = not significant (*p* > 0.05). *BMI* Body mass index, *PLI* Plaque index, *BOP* Bleeding on probing, *PPD* Probing pocket depth, *CAL* Clinical attachment level

Mean and SD values calculated for the demographic variables and periodontal parameters of all four study groups, are presented in Table 1. Of the 120 patients retrospectively analysed in this study, demographic data distributions were determined for sex (25 female, 95 male), age range (35–75 years), with all participants possessing BMI scores of < 25 kg/m^2^. The mean age, BMI values, and gender distribution of participants were not significant different between each group (*p* > 0.05). The sample site analysis of periodontal parameters, PLI, BOP, PPD and CAL, were demonstrated to be all statistically greater for the P and AS-P groups with and without diagnosed CHD, compared to the healthy periodontium participants, C and AS-C (all *p* < 0.001). The group comparisons for all variables are presented in Fig. 2.

**Table 1:** Demographic characteristics and clinical periodontal parameters of the control (C), generalized periodontitis (P), CHD and clinically healthy periodontium (AS-C); and CHD with generalized periodontitis (AS-P) groups

	**Control (C) (*n* = 30)**	**Generalized Periodontitis (P) (*n* = 30)**	**CHD and clinically healthy periodontium (AS-C) (*n* = 30)**	**CHD with generalized periodontitis (AS-P) (*n* = 30)**	**Group *p* Value**
Age	54.0 (7.6)	54.8 (7.5)	58.1 (7.0)	57.8 (5.9)	0.061
BMI	21.35 (1.68)	21.18 (1.27)	21.17 (1.27)	21.42 (2.17)	0.529
Gender					
Male (*n*, %)	24 (80.0%)	24 (80.0%)	24 (80.0%)	23 (76.7%)	1.00
Female (*n*, %)	6 (20.0%)	6 (20.0%)	6 (20.0%)	7 (23.3%)	
PLI	12.83 (2.87)	59.50 (3.66)	19.17 (2.63)	60.80 (3.50)	< 0.001
BOP (%)	9.03 (2.54)	70.30 (4.98)	8.93 (2.42)	69.27 (3.71)	< 0.001
PPD (mm)	0.00 (0.00)	4.88 (1.60)	0.00 (0.00)	7.23 (1.74)	< 0.001
CAL (mm)	0.00 (0.00)	6.56 (1.60)	0.00 (0.00)	8.00 (1.50)	< 0.001

Data expressed as the mean ± SD (in brackets). *BMI* Body mass index, *PLI* Plaque index, *BOP* Bleeding on probing, *PPD* Probing pocket depth, *CAL* Clinical attachment level. One-way ANOVA with post-hoc Tukey test, except gender comparisons performed by Chi Square test. Statistically significant differences between the study groups considered at *p* < 0.05

### Salivary IL-1β protein levels

**Fig. 3:**
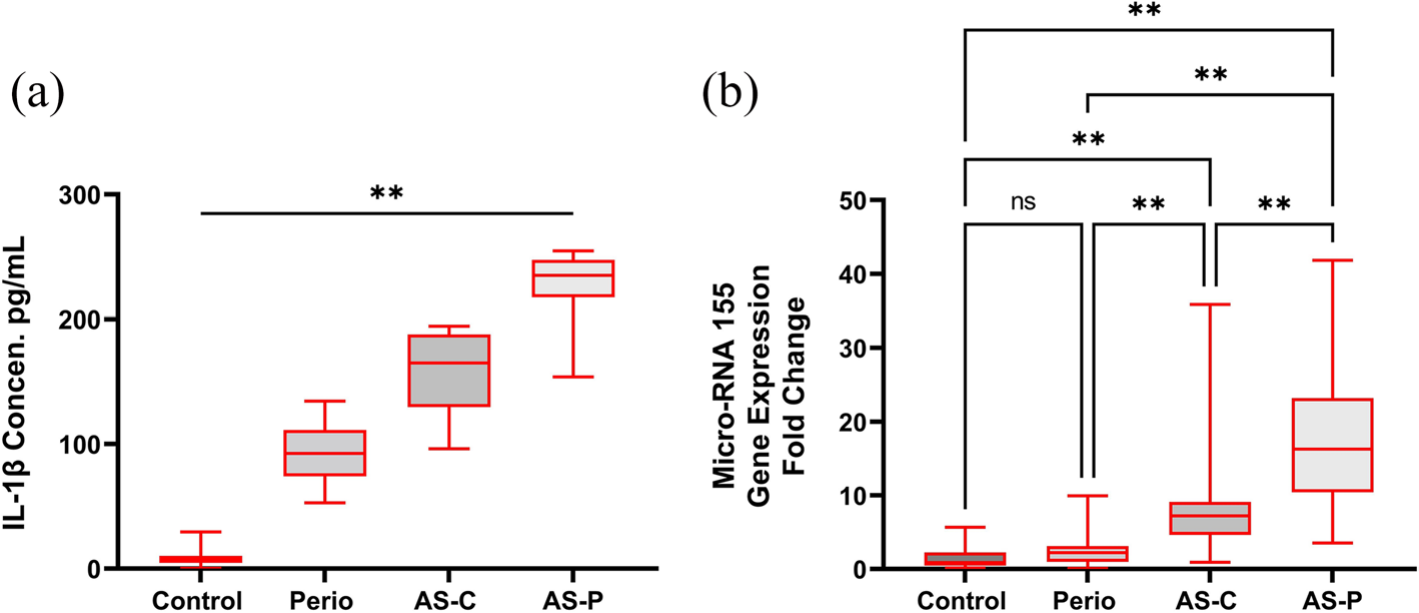
Levels of (**a**) IL-1β (pg/mL) in saliva samples and (**b**) fold changes in miR-155 expression in whole blood samples, collected from control (C), generalized periodontitis (P), CHD and clinically healthy periodontium (AS-C); and CHD with generalized periodontitis (AS-P) groups. Data are expressed as the mean ± SD. Comparisons made using one-way Analysis of Variance (ANOVA), with post-hoc Tukey test. **p < 0.01 versus control group (C)

Mean and SD values obtained for the quantification of salivary IL-1β levels, are shown in Fig. 3a. Overall, mean IL-1β detected in the saliva of participants with CHD (AS-P, 194.15 pg/mL; AS-C, 123.29 pg/mL) and periodontitis alone (P, 78.43 pg/mL), were significantly higher than the control group (C, 5.94 pg/mL) (F value = 17.97, *p* < 0.001).

### miR-155 expression levels in whole blood

Mean and SD values obtained for the quantification of fold-changes in miR-155 expression detectable in whole blood, are shown in Fig. 3b. Mean fold changes in miR-155 expression were determined to be significantly higher in participants with CHD (AS-P, 18.30; AS-C, 9.30) and periodontitis alone (P; 4.30), compared to the control group (C, 1.30) (F value = 7.74, *p* < 0.001).

### Diagnostic accuracy of salivary IL-1β and whole blood miR-155 biomarkers

**Fig. 4:**
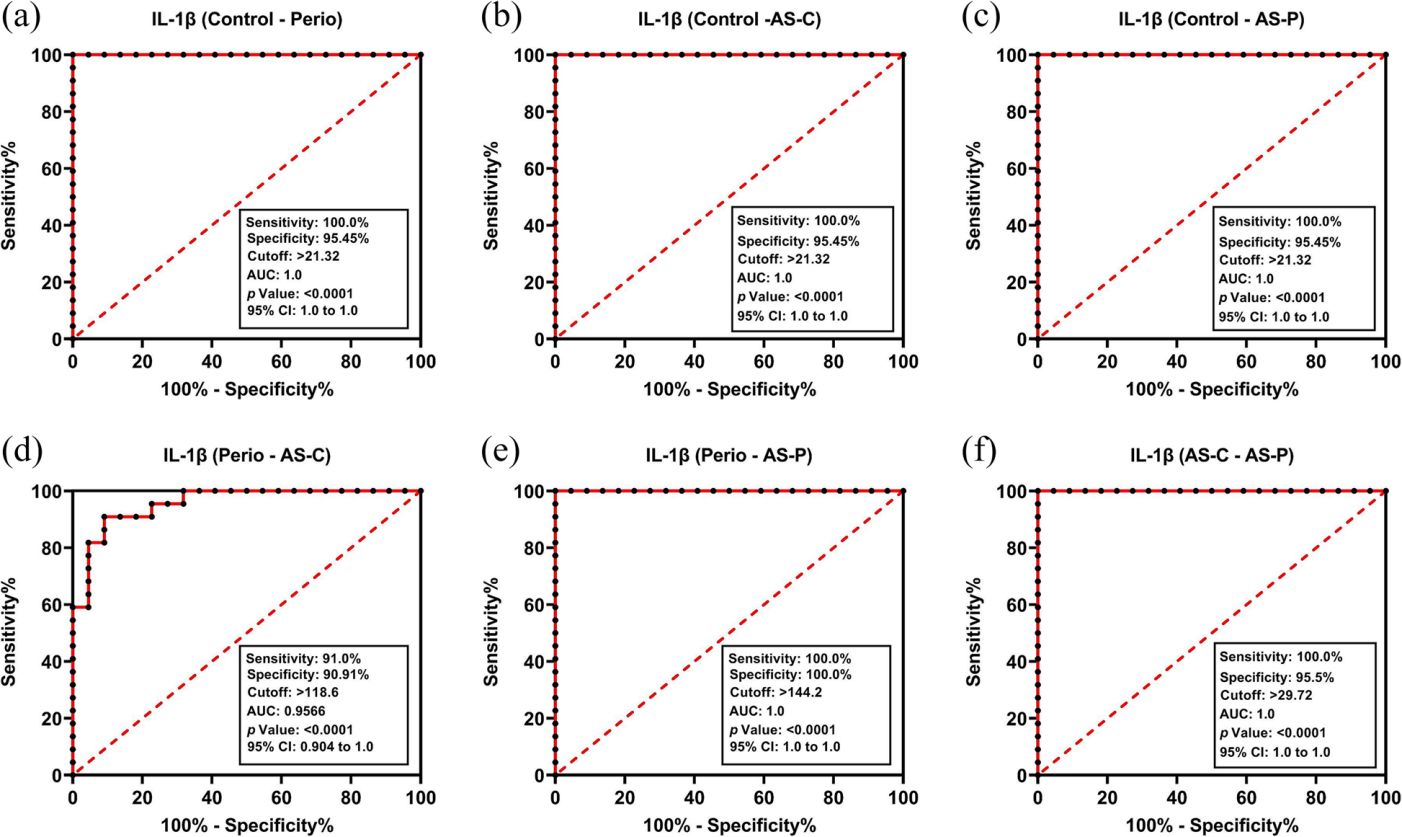
Receiver operating characteristic (ROC) curves differentiating healthy from generalized periodontitis patients with and without CHD, by comparing salivary IL-1β levels between the control group (C) versus (**a**) generalized periodontitis (P) patients, (**b**) CHD and clinically healthy periodontium (AS-C) patients; and (**c**) CHD with generalized periodontitis (AS-P) patients. Further ROC curves distinguishing between generalized periodontitis (P) patients versus (**d**) CHD and clinically healthy periodontium (AS-C) patients, (**e**) CHD with generalized periodontitis (AS-P) patients; in addition to (**f**) between CHD and clinically healthy periodontium (AS-C) patients and CHD with generalized periodontitis (AS-P) patients. Data were obtained by logistic regression analysis (all *p* < 0.0001)

The diagnostic potential (sensitivity and specificity) of each biomarker to discriminate between healthy controls versus periodontitis and non-periodontitis patients with/without CHD, were estimated using ROC curves and corresponding AUC analyses. The accuracy of salivary IL-1β as a discriminatory biomarker of periodontitis and CHD, were illustrated by the ROC curves generated for each study group (Fig. 4a-f). These showed high accuracy for P, AS-C, and AS-P, since AUC values were 1.00 (all *p* < 0.0001). Therefore, these results demonstrated a high sensitivity of 100.0%, coupled with a specificity of 95.5%, at a cut-off point of > 21.32 arbitrary units (a.u.) for all CHD (AS-P, AS-C) and periodontitis alone (P) groups, compared to the control group (C). These results also demonstrated a high sensitivity of ≥ 91.0%, coupled with a specificity of ≥ 90.9% at a cut-off point of > 118 a.u. and AUC accuracy of ≥ 0.9566, for all CHD (AS-C, AS-P) compared to periodontitis alone (P) groups (all *p* < 0.0001, Fig. 4d-e). Similarly, a comparison between AS-C and AS-P demonstrated a high sensitivity of 100.0%, coupled with a specificity of 95.5% at a cut-off point of > 29.72 a.u and AUC accuracy of 1.00 (*p* < 0.0001, Fig. 4f).

**Fig. 5:**
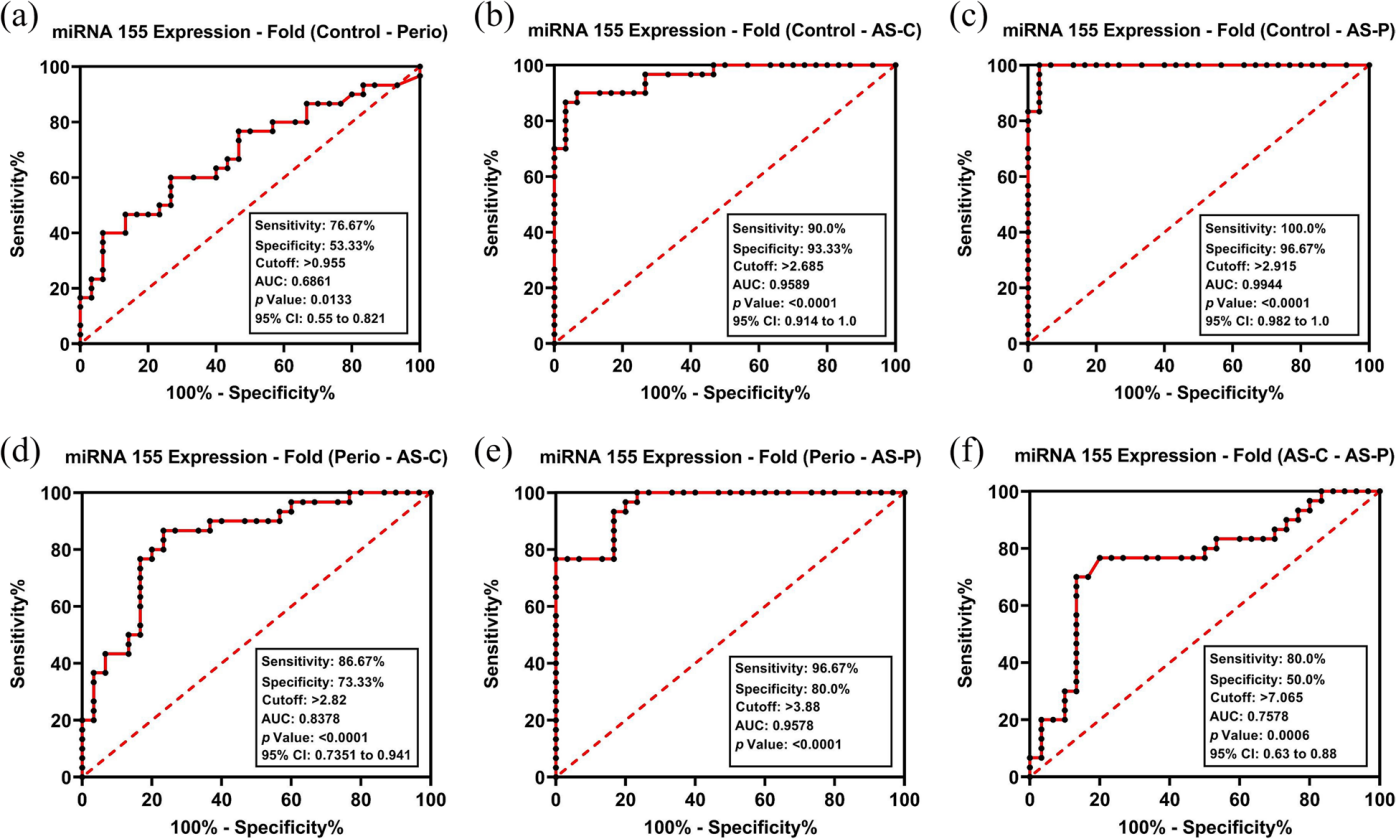
Receiver operating characteristic (ROC) curves differentiating healthy from generalized periodontitis patients with and without CHD, by comparing whole blood miR-155 levels between the control group (C) versus (**a**) generalized periodontitis (P) patients, (**b**) CHD and clinically healthy periodontium (AS-C) patients; and (**c**) CHD with generalized periodontitis (AS-P) patients. Further ROC curves distinguishing between generalized periodontitis (P) patients versus (**d**) CHD and clinically healthy periodontium (AS-C) patients, (**e**) CHD with generalized periodontitis (AS-P) patients; in addition to (**f**) between CHD and clinically healthy periodontium (AS-C) patients and CHD with generalized periodontitis (AS-P) patients. Data were obtained by logistic regression analysis (all *p* < 0.001, except (a) miR-155 level comparison between control group (C) and generalized periodontitis (P) patients, *p* < 0.05)

The accuracy of whole blood miR-155 expression as a biomarker of periodontitis and CHD, was further shown by the ROC curves obtained for each group (Fig. 5a-f), which displayed a high accuracy for P, AS-C, and AS-P overall, with AUC values of 0.6861, 0.9589 and 0.9944, respectively (*p* < 0.0001–0.05). High sensitivity was further demonstrated by miR-155 expression levels, with values of 76.7%, 90.0%, and 100.0% for these groups, respectively. miR-155 expression specificity was determined with values of 53.3%, 93.3% and 96.7%, at a cut-off point of > 0.955, > 2.685 and > 2.915 a.u. for the periodontitis alone (P) and CHD (AS-P, AS-C) groups, respectively; versus the control group (C). These results also collectively demonstrated a high sensitivity of ≥ 88.7%, coupled with a specificity of ≥ 73.3% at a cut-off point of > 2.820 a.u. and AUC accuracy of ≥ 0.8378, for all CHD (AS-C, AS-P) compared to periodontitis alone (P) groups (both *p* < 0.0001, Fig. 5d-e). Similarly, a comparison between AS-C and AS-P demonstrated a high sensitivity of 80.0%, coupled with a specificity of 50.0% at a cut-off point of > 7.065 a.u and AUC accuracy of 0.7578 (*p* < 0.001, Fig. 5f). Based on the ROC curve data, a summary comparison of biomarker diagnostic properties in discriminating healthy control and periodontitis patients, with or without CHD, such as sensitivity, specificity and cut-off points, are shown in Table 2.

**Table 2:** Summary comparison of biomarker diagnostic properties for biomarker (miR-155, IL-1β) thresholds between control (C), generalized periodontitis (P), CHD and clinically healthy periodontium (AS-C); and CHD with generalized periodontitis (AS-P) groups

Biomarker	Group Comparisons	Sensitivity	Specificity	Youden Index	AUC	95% CI	Cut-Off Point	*p* Value
**IL-1β (pg/mL)**	C group vs. P group	100.0%	95.5%	0.95	1.00	1.000–1.000	> 21.32	< 0.0001
	C group vs. AS-C group	100.0%	95.5%	0.95	1.00	1.000–1.000	> 21.32	< 0.0001
	C group vs. AS-P group	100.0%	95.5%	0.95	1.00	1.000–1.000	> 21.32	< 0.0001
	P group vs. AS-C group	91.0%	90.9%	0.82	0.956	0.904–1.000	> 118.6	< 0.0001
	P group vs. AS-P group	100.0%	100.0%	1.00	1.00	1.000–1.000	> 144.2	< 0.0001
	AS-C group vs. AS-P group	100.0%	95.5%	0.96	1.00	1.000–1.000	> 29.72	< 0.0001
**miR-155(RQ)**	C group vs. P group	76.7%	53.3%	0.30	0.686	0.550–0.821	> 0.955	< 0.0001
	C group vs. AS-C group	90.0%	93.3%	0.83	0.958	0.914–1.000	> 2.685	< 0.0001
	C group vs. AS-P group	100.0%	97.7%	0.98	0.994	0.982–1.000	> 2.915	< 0.0001
	P group vs. AS-C group	86.7%	73.3%	0.60	0.837	0.735–0.941	> 2.820	< 0.0001
	P group vs. AS-P group	96.7%	80.0%	0.77	0.957	0.915–1.000	> 3.880	< 0.0001
	AS-C group vs. AS-P group	80.0%	50.0%	0.30	0.757	0.630–0.880	> 7.065	< 0.0006

*AUC* Area under the curve, *95% CI* 95% Confidence interval. McNemar test. Statistically significant differences between respective study groups considered at *p* < 0.05

#### Correlations between miR-155, IL-1β, demographic data and clinical variables

**Fig. 6:**
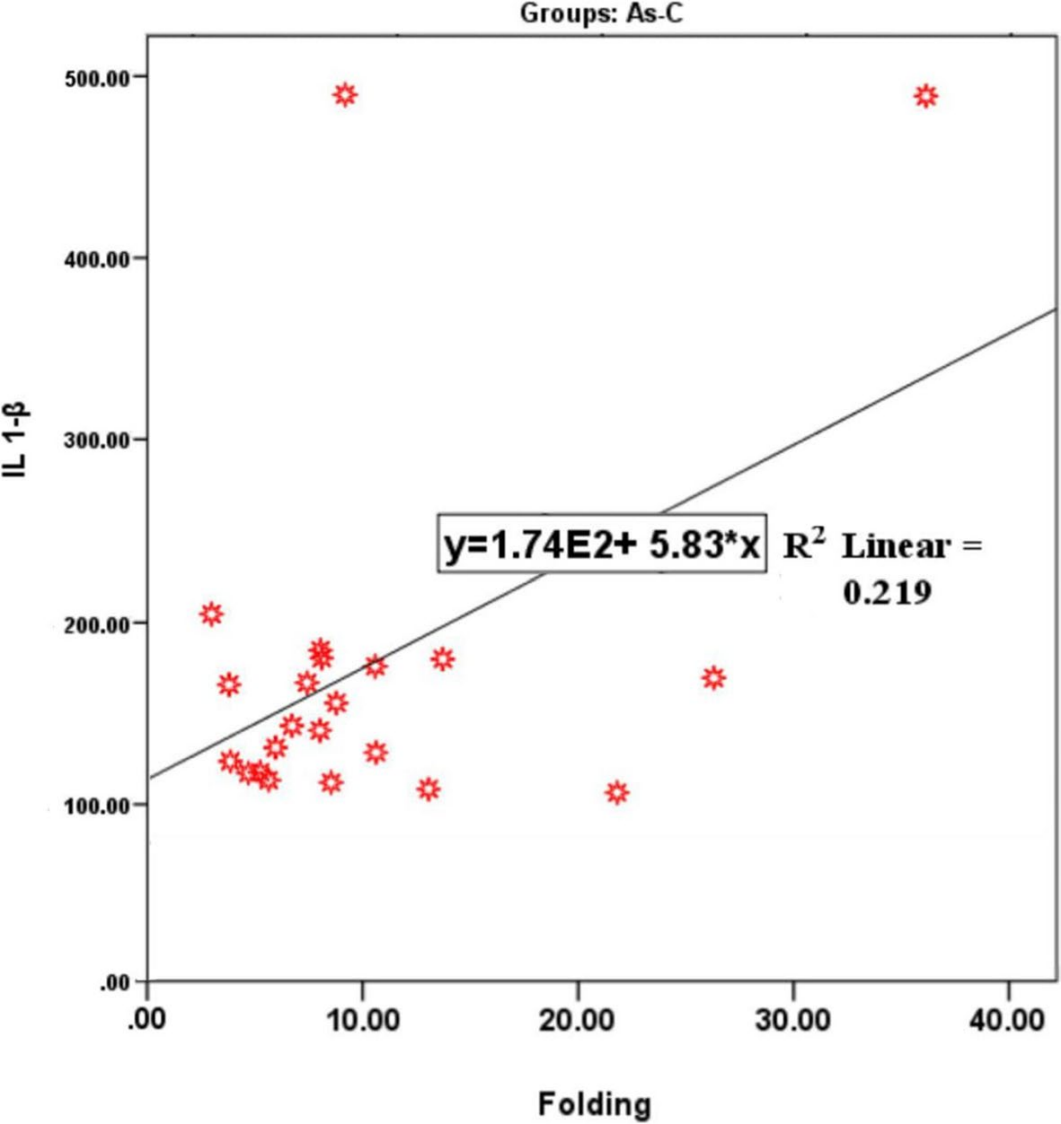
Linear regression analysis demonstrating a significant and positive correlation between salivary IL-1β protein levels and the fold changes in miR-155 expression within the AS-C group (*r* = 0.468, *p* = 0.028, R^2^ = 0.219)

Pearson correlation test coefficients (r) calculated between whole blood miR-155 expression and study participant age, BMI, gender, clinical parameters and salivary IL-1β protein levels for each group; are shown in Table 3. The data indicated that significant positive correlations between miR-155 expression and the PPD of the P group (*r* = 0.395, *p *= 0.031), CAL of the AS-P group (*r* = 0.440, *p* = 0.015); and IL-1β levels of the AS-C group (*r *= 0.468, *p* = 0.028; linear regression analysis in Fig. 6, R^2^ = 0.219). As a result, a significant correlation between miR-155 expression and IL-1β levels for the AS-C group was shown by linear regression analysis (Fig. 6, R^2^ = 0.219), although only a relatively weak positive correlation was identified.

**Table 3:** Pearson correlation test coefficients (r) calculated between whole blood miR-155 expression and study participant age, BMI, gender, clinical parameters and salivary IL-1β protein levels, for control (C), generalized periodontitis (P), CHD and clinically healthy periodontium (AS-C); and CHD with generalized periodontitis (AS-P) groups

	**Control (C) (*n* = 30)**		**Generalized Periodontitis (P) (*n* = 30)**		**CHD and clinically healthy periodontium (AS-C) (*n* = 30)**		**CHD with generalized periodontitis (AS-P) (*n* = 30)**	
	***r* Value**	***p* Value**	***r* Value**	***p* Value**	***r* Value**	***p* Value**	***r* Value**	***p* Value**
Age	0.090	0.637	0.036	0.849	0.183	0.332	0.243	0.195
BMI	0.134	0.479	-0.308	0.098	0.175	0.356	0.017	0.930
Gender	0.023	0.906	0.324	0.081	0.209	0.267	0.000	0.999
PLI	-0.073	0.703	-0.039	0.836	-0.009	0.963	0.097	0.609
BOP (%)	-0.022	0.907	0.319	0.085	-0.091	0.634	0.016	0.935
PPD (mm)	-	-	0.395	**0.031**	-	-	0.327	0.077
CAL (mm)	-	-	0.099	0.602	-	-	0.440	**0.015**
IL-1β (pg/mL)	-0.027	0.907	0.087	0.700	0.468	**0.028**	0.136	0.545

*r value* Correlation test coefficient, *BMI* Body mass index, *PLI* Plaque index, *BOP* Bleeding on probing, *PPD* Probing pocket depth, *CAL* Clinical attachment level. Pearson correlation tests and multiple linear regression analyses. Statistically significant differences between respective study groups considered at *p* < 0.05, with significant p values denoted in bold

## Discussion

Although our understanding of miRs and their roles in immuno-inflammatory responses, pathology and healing associated with periodontal disease are ever-emerging [23–25, 46], to our knowledge, this is the first study to quantify miR-155 expression in whole blood and attempt to correlate these levels with patient CHD, with and without additional clinical diagnoses of periodontitis. Furthermore, this study is the first to examine the correlation between whole blood miR-155 expression and salivary IL-1β protein levels, to ascertain miR-155 validity as an accurate biomarker of chronic inflammation and host tissue damage associated with periodontitis and atherosclerotic plaque formation.

Both periodontitis and CHD comprise complicated processes involving local chronic inflammation and systemic tissue damage. As miRs play a crucial role in the regulation of several physiological and pathological processes, these have further been implicated in facilitating the onset and progression of periodontitis and CHD [19–21, 24, 25, 27]. The most significant finding of this study was that fold changes in whole blood miR-155 expression were higher in participants with generalized periodontitis (P), P with CHD (AS-P) and CHD alone (AS-C), compared to the control patient cohort (C). Therefore, higher miR-155 expression in disease patients corroborate most previous reports in periodontitis and CHD patients [29–34]. Although miR-155 upregulation is a protective response to periodontal inflammation and atherosclerosis, elevated miR-155 levels would potentially contribute to the initiation and progression of periodontitis and CHD via numerous mechanisms, including the promotion of osteoclastogenesis and M1 macrophage differentiation; the main mechanisms associated with periodontal bone loss and atherosclerotic plaque formation [21, 24, 32]. Enhanced miR-155 levels with CHD severity could additionally increase pro-inflammatory mediator generation, causing endothelial cell dysfunction and apoptosis; and altering toll-like receptor (TLR), angiotensin II and Janus kinase 2/signal transducer and activator of transcription 3 (JAK2/STAT3) signalling [47–49], whilst further disrupting other key responses in smooth muscle cells [50, 51].

Fold changes in miR-155 expression also exhibited significant positive correlations between miR-155 expression and the PPD of the P group, CAL of the AS-P group; and salivary IL-1β levels of the AS-C group. Therefore, a correlation may exist between periodontal tissues destruction, CHD and elevated IL-1β levels in saliva; and provide evidence for miR-155 having a role in modulating periodontitis in patients, with and without clinically diagnosed CHD. IL-1β production increases when microbial components and oxidised LDLs stimulate host immuno-inflammatory and connective tissue cells, with IL-1β recognized as major modulator of osteoclastogenesis, a primary cause of periodontal tissue damage and endothelial cell dysfunction during CHD [10, 32, 52]. Levels of both miR-155 and IL-1β could differentiate between healthy, generalized periodontitis and CHD patients with high accuracy, based on ROC curve representation of the combined sensitivity, specificity and cut-off analysis for each patient group.

Two of the demographic variables that this present study assessed, age and BMI, were found to be relevant to all four study groups. However, the mean value of BMI was within the ordinary range for all groups to prevent confounding effects. The AS groups had higher mean BMI than the P and C groups. This is because the increased BMI contributes directly to cardiovascular risk factors and associates with atherosclerotic plaque formation [53].

Periodontal parameters, PI, BOP, PPD and CAL, were all higher in the AS-P group consisting of participants with diagnosed periodontitis and CHD, versus other study groups. The findings of the Pearson correlation coefficients supported these conclusions, with positive correlations between miR-155 expression levels and the various clinical periodontal parameters assessed, and salivary IL-1β protein levels. The findings of the liner regression, similarly, indicated a significant and positive correlation between miR-155 and IL-1β levels, particularly in the AS-C group. Similar findings have been reported previously [41, 42]. However, despite numerous studies reporting correlations between periodontal inflammatory markers, atherosclerosis and CHD, such correlations are not always robust. Nonetheless, such studies propose that these events are a consequence of sub-gingival periodontal pathogen accumulation and the release of bacteria and their products from established plaque biofilms into the bloodstream, which can cause bacteraemia and/or endo-toxaemia. Coupled with the concurrent stimulation of host immuno-inflammatory mechanisms adjacent to blood vessel walls, these responses create a pro-thrombotic environment, leading to the onset and progression of atherosclerosis and CHD [54, 55]. Indeed, atherosclerosis and CHD patients have live bacteria and significantly higher levels of IL-1β and other pro-inflammatory markers, which directly stimulates atheroma formation and can worsen periodontal destruction [42, 56, 57].

The present study is not without limitations. Although the use of random sampling for participant selection may have overlooked some suitable candidates, a sequential case–control strategy was used to enrol all the participants to minimise bias. Furthermore, although this is a pilot study, a larger patient cohort sample size may now be warranted to further investigate the relationships between miR-115 expression levels with periodontal and CHD status. This would be aided by the employment of a CHD severity score, such as the Gensini score, as it requires more participants and a longer time commitment. Thus, in addition to miR-155 expression being a potential biomarker that can be used to distinguish between periodontitis and CHD, it may also provide insights into the mutual correlations between the two diseases. Therefore, although miR-155 is a promising therapeutic modality in the prevention and treatment of CHD-associated periodontal disease, further in-depth longitudinal and interventional studies are needed to better understand its complex involvement, such as the evaluation of miR-155 expression at different stages of periodontitis, which may provide a clearer understanding on how miR-155 levels change during periodontitis progression.

To conclude, it is now well-established that miR-155 regulates normal immuno-inflammatory responses via its pro- and anti-inflammatory properties, whilst miR-155 expression is dysregulated during uncontrolled chronic inflammation, which contributes to the pathologies of periodontitis and atherosclerosis. Thus, as evident herein with the elevated levels of miR-155 in the whole blood of P, AS-C and AS-P patient groups, compared to the C group; elevated miR-155 levels in periodontitis and CHD patients are often accompanied by higher salivary IL-1β levels and changes in clinical periodontal parameters. Consequently, miR-155 may be developed as an accurate inflammatory biomarker for the diagnosis of periodontitis and atherosclerosis; and even used to predict the severity of periodontitis and CHD in future, thereby offering improved early detection strategies and treatment intervention opportunities for both of these significant and widespread conditions.

### Supplementary Information

**Additional file 1. **STROBE checklist of items that should be included in reports of cross-sectional case-control observational studies with detailed referencing of requirements to the text of the paper

## Data Availability

All data and materials used are available by the corresponding authors on reasonable request.

## Abbreviations

3UTR: 3’ Untranslated region
ANOVA: Analysis of Variance
AS-C: Patients with CHD and clinically healthy periodontium
AS-P: Patients with CHD with generalized periodontitis
a.u.: Arbitrary units
AUC: Area under the curve
BMI: Body mass index
BOP: Bleeding on probing
C: Controls
CAL: Clinical attachment loss
cDNA: Complimentary DNA
CHD: Coronary heart disease
CRP: C-reactive protein
ECG: Electrocardiogram
ELISA: Enzyme-linked immunosorbent assay
HDL: High-density lipoprotein
ICC: Intra-class correlation coefficients
IL-1β: Interleukin-1β
JAK2: Janus kinase 2
LDL: Low-density lipoprotein
MiR: Micro ribonucleic acids
miR-155: MicroRNA‐155
NSTEMI: Non-ST-elevation myocardial infraction-heart attack
P: Generalized periodontitis
PLI: Plaque index
PPD: Probing pocket depth
qRT-PCR: Quantitative real time polymerase chain reaction
ROC: Receiver operating characteristic
RQ: Relative fold changes
RT-PCR: Reverse transcription polymerase chain reaction
SD: Standard deviation
STAT3: Signal transducer and activator of transcription 3
STEMI: ST-elevation myocardial infraction-heart attack
TLR: Toll-like receptor
