# Correction: MicroRNA-155 (miR-155) as an accurate biomarker of periodontal status and coronary heart disease severity: a case–control study

**DOI:** 10.1186/s12903-024-04112-0

**Published:** 2024-03-19

**Authors:** Zina A. Daily, Batool Hassan Al-Ghurabi, Ahmed Makki A. Al-Qarakhli, Ryan Moseley

**Affiliations:** 1 Department of Periodontics, College of Dentistry, University of Baghdad, Baghdad, Iraq; 2 Department of Periodontics, College of Dentistry, University of Al-Ameed, Karbala, Iraq; 3 Department of Basic Science, College of Dentistry, University of Baghdad, Baghdad, Iraq; 4 Department of Oral Diagnosis, College of Dentistry, University of Anbar, Ramadi, Iraq; 5 Disease Mechanisms Group, School of Dentistry, College of Biomedical and Life Sciences, Cardiff University, Cardiff, UK

**Correction to: BMC Oral Health (2023) 23:868**

10.1186/s12903-023-03584-w.

In this article [1], the author’s name Batool Hassan Al‑Ghurabi was incorrectly written as Batool Hassan Al‑Ghurabei.

The original article has been corrected.

