# Supplementary material for: MicroRNA‐155 (miR-155) as an accurate biomarker of periodontal status and coronary heart disease severity: a case–control study
Source: BMC Oral Health. 2023 Nov 16;23:868. doi: 10.1186/s12903-023-03584-w (PMC10652601; doi:10.1186/s12903-023-03584-w)
Supplement: Supplementary file 1 — Additional file 1. STROBE checklist of items that should be included in reports of cross-sectional case-control observational studies with detailed referencing of requirements to the text of the paper [file 12903_2023_3584_MOESM1_ESM.doc]

STROBE checklist of items that should be included in reports of cross-sectional case-control observational studies with detailed referencing of requirements to the text of the paper.

|  | | Item No | Recommendation |
| --- | --- | --- | --- |
| **Title and abstract** | | 1 | (*a*) Indicate the study’s design with a commonly used term in the title or the abstract  Case-control study as stated in the Abstract on page 2 and Methods on page 4. |
|  |  |  | (*b*) Provide in the abstract an informative and balanced summary of what was done and what was found  Provided in Abstract on page 2. |
| Introduction | | | |
| Background/rationale | | 2 | Explain the scientific background and rationale for the investigation being reported  Included in the Background on pages 4-6. |
| Objectives | | 3 | State specific objectives, including any prespecified hypotheses  Included in the Background on page 6. |
| Methods | | | |
| Study design | | 4 | Present key elements of study design early in the paper  Included in the Materials and methods on pages 6-7. |
| Setting | | 5 | Describe the setting, locations, and relevant dates, including periods of recruitment, exposure, follow-up, and data collection  Included in the Methods on pages 6-8. |
| Participants | | 6 | (*a*) *Cohort study*—Give the eligibility criteria, and the sources and methods of selection of participants. Describe methods of follow-up  *Case-control study*—Give the eligibility criteria, and the sources and methods of case ascertainment and control selection. Give the rationale for the choice of cases and controls  *Cross-sectional study*—Give the eligibility criteria, and the sources and methods of selection of participants  Included in the Materials and methods on pages 7-9. |
|  |  |  | (*b*) *Cohort study*—For matched studies, give matching criteria and number of exposed and unexposed  *Case-control study*—For matched studies, give matching criteria and the number of controls per case  Included in the Materials and methods on pages 8-10. |
| Variables | | 7 | Clearly define all outcomes, exposures, predictors, potential confounders, and effect modifiers. Give diagnostic criteria, if applicable  Included in the Materials and methods on pages 7 and 9-11. |
| Data sources/ measurement | | 8* | For each variable of interest, give sources of data and details of methods of assessment (measurement). Describe comparability of assessment methods if there is more than one group  Included in the Materials and methods on pages 8-11. |
| Bias | | 9 | Describe any efforts to address potential sources of bias  Addressed in the limitations paragraph in the Discussion on page 18. |
| Study size | | 10 | Explain how the study size was arrived at  Included in the Materials and methods on pages 6-7. |
| Quantitative variables | | 11 | Explain how quantitative variables were handled in the analyses. If applicable, describe which groupings were chosen and why  Included in the Materials and methods on pages 10-11. |
| Statistical methods | | 12 | (*a*) Describe all statistical methods, including those used to control for confounding  Included in the Materials and methods on page 12. |
|  |  |  | (*b*) Describe any methods used to examine subgroups and interactions  Included in the Materials and methods on page 12. |
|  |  |  | (*c*) Explain how missing data were addressed  Included in the Materials and methods on page 12. |
|  |  |  | (*d*) *Cohort study*—If applicable, explain how loss to follow-up was addressed  *Case-control study*—If applicable, explain how matching of cases and controls was addressed Included in the Materials and methods on page 8.  *Cross-sectional study*—If applicable, describe analytical methods taking account of sampling strategy |
|  |  |  | (*e*) Describe any sensitivity analyses Not applicable. |
| Results | | | |
| Participants | 13* | (a) Report numbers of individuals at each stage of study—eg numbers potentially eligible, examined for eligibility, confirmed eligible, included in the study, completing follow-up, and analysed  Included in the Materials and methods on page 8. | |
|  |  | (b) Give reasons for non-participation at each stage  Included in the Materials and methods on page 8. | |
|  |  | (c) Consider use of a flow diagram  Included in the Materials and methods as Fig. 1. | |
| Descriptive data | 14* | (a) Give characteristics of study participants (eg demographic, clinical, social) and information on exposures and potential confounders  Included in the Materials and methods on pages 7-8. | |
|  |  | (b) Indicate number of participants with missing data for each variable of interest  Not applicable. | |
|  |  | (c) *Cohort study*—Summarise follow-up time (eg, average and total amount)  Not applicable. | |
| Outcome data | 15* | *Cohort study*—Report numbers of outcome events or summary measures over time | |
|  |  | *Case-control study—*Report numbers in each exposure category, or summary measures of exposure Included in the Materials and methods on page 8. | |
|  |  | *Cross-sectional study—*Report numbers of outcome events or summary measures | |
| Main results | 16 | (*a*) Give unadjusted estimates and, if applicable, confounder-adjusted estimates and their precision (eg, 95% confidence interval). Make clear which confounders were adjusted for and why they were included  Included in the Results on pages 13-15 and in Tables 1-3. | |
|  |  | (*b*) Report category boundaries when continuous variables were categorized  Included in the Results on pages 13-15, in Tables 1-3 and in Fig. 2-6. | |
|  |  | (*c*) If relevant, consider translating estimates of relative risk into absolute risk for a meaningful time period. Not applicable. | |
| Other analyses | 17 | Report other analyses done—eg analyses of subgroups and interactions, and sensitivity analyses  Included in the Materials and methods on page 12. | |
| Discussion | | | |
| Key results | 18 | Summarise key results with reference to study objectives  Included in the Discussion on pages 15-16. | |
| Limitations | 19 | Discuss limitations of the study, taking into account sources of potential bias or imprecision. Discuss both direction and magnitude of any potential bias  Included in the Discussion on page 18. | |
| Interpretation | 20 | Give a cautious overall interpretation of results considering objectives, limitations, multiplicity of analyses, results from similar studies, and other relevant evidence  Included in the Discussion on pages 15-18. | |
| Generalisability | 21 | Discuss the generalisability (external validity) of the study results  Included in the Discussion on pages 18-19. | |
| Other information | | | |
| Funding | 22 | Give the source of funding and the role of the funders for the present study and, if applicable, for the original study on which the present article is based  Provided in the text on page 22. | |
